# Analysis of Citrus Bioflavonoid Content and Dipeptidyl Peptidase-4 Inhibitory Potential of Commercially Available Supplements

**DOI:** 10.3390/molecules27154741

**Published:** 2022-07-25

**Authors:** Ankit Gupta, Hayder A. Al-Aubaidy, Christian K. Narkowicz, Herbert F. Jelinek, David S. Nichols, John R. Burgess, Glenn A. Jacobson

**Affiliations:** 1 School of Medicine, University of Tasmania, Hobart, TAS 7000, Australia; ankit.gupta@utas.edu.au (A.G.); c.narkowicz@utas.edu.au (C.K.N.); j.burgess@utas.edu.au (J.R.B.); glenn.jacobson@utas.edu.au (G.A.J.); 2 Department of Microbiology, Anatomy, Physiology & Pharmacology, La Trobe University, Bundoora, VIC 3086, Australia; 3 Department of Biomedical Engineering and Health Engineering Innovation Center, Khalifa University, Abu Dhabi 127788, United Arab Emirates; herbert.jelinek@ku.ac.ae; 4 Biotechnology Center, Khalifa University, Abu Dhabi 127788, United Arab Emirates; 5 Central Science Laboratory, University of Tasmania, Hobart, TAS 7005, Australia; d.nichols@utas.edu.au; 6 Department of Diabetes & Endocrinology, Royal Hobart Hospital, Hobart, TAS 7000, Australia

**Keywords:** bioflavonoid, citrus, dipeptidyl peptidase-4 (DPP-4), rutin, supplements, flavonoids, hesperidin

## Abstract

Citrus bioflavonoids are polyphenolic plant-derived pigments found in high levels in oranges, lemons, grapefruits and other citrus fruits. The three most abundant types of citrus bioflavonoids are hesperidin, naringenin and eriocitrin. Citrus bioflavonoids have long been known to possess powerful free radical-scavenging properties and cardioprotective effects. The study involved the analysis of 10 commercially available citrus bioflavonoid supplements from three different countries: Australia, the United States and Canada. The supplements were tested for their citrus bioflavonoid content which varied from 0.8 to 33.3% *w*/*w*. The daily bioflavonoid dose varied from 19 mg to 560 mg. Hesperidin was the major citrus bioflavonoid in nine out of ten supplements. One supplement was found to contain less than 10% of the quantity of rutin claimed to have been added. The DPP-4 inhibitory potential, compared through an estimation of rutin equivalence, ranged from 1.9 mg to 400 mg per day. This data highlights the variability between the supplements in their potential to inhibit DPP-4 for subsequent health benefits.

## 1. Introduction

Bioflavonoids are naturally occurring compounds present in various fruits, vegetables, chocolate and beverages such as tea and wine. Bioflavonoids can be divided into different subclasses according to the chemical structure of their tricyclic ring system. The most important dietary subclasses include flavanols, flavonols, flavanones, anthocyanins, flavones and isoflavones [1]. The main subclass present in citrus fruits is flavanones, and this includes the compounds eriocitrin, eriodictyol, hesperidin, hesperetin, naringin and naringenin. Citrus bioflavonoid supplements are manufactured in multiple countries using citrus by-products from fruit processing, such as juice production. Different supplements in the market provide a wide variety of claimed bioflavonoid content. Product labels typically describe the content of citrus bioflavonoid extract or complex, but do not provide information on the actual content of bioflavonoids present in each supplement. It is important to know the actual bioflavonoid content because the potential effectiveness of a supplement will depend on the quantity of active ingredients per dose. There is currently no information available from manufacturers or from research studies that describes the flavonoid profile or content of commercially available citrus bioflavonoid supplements.

Citrus bioflavonoid supplements have the potential to improve glycaemic regulation in diabetic and prediabetic individuals. Dipeptidyl peptidase-4 (DPP-4) is an enzyme expressed as a glycoprotein in both a transmembranous form and a soluble form within plasma. DPP-4 serves a pivotal role in maintaining the activity of the incretin hormones which include GIP (glucose insulinotropic polypeptide) and GLP-1 (glucagon-like peptide) [2]. We have previously shown that citrus bioflavonoids exhibit in vitro inhibition of DPP-4 [3]. Since, the DPP-4 inhibitory activity is concentration-dependent, the potential of citrus bioflavonoid supplements to affect glycaemic control will depend on their actual bioflavonoid content and the daily dose of supplement.

In Australia, nutritional supplements are regulated as medicines. They must be approved by the Therapeutic Goods Administration (TGA) prior to entering the market and must be manufactured in a facility approved by the TGA under GMP guidelines. Canada has a similar system which is regulated by the Natural and Non-prescription Health Products Directorate (NNHPD). By contrast, in the United States (US), nutritional supplements are not regulated as medicines but the manufacturer is expected to meet the requirements of the Food and Drug Administration (FDA) with respect to labelling and lack of adulteration. Products do not require approval from the FDA before entering the market. As a consequence, there is greater potential for US-based manufacturers to produce supplements of poor quality.

This study aimed to survey 10 different commercially available supplements manufactured in the United States, Australia and Canada, for content variation of citrus bioflavonoids and to compare the DPP-4 inhibitory potential of these supplements.

## 2. Materials and Methods

### 2.1. Supplements

Convenience sampling was used to select 10 different citrus bioflavonoid supplements, labelled CBF-1 to CBF-10, from products available over the counter or online in Australia. Supplements were manufactured in Australia (AUS), the United States of America (USA) and Canada (CAN) and had label claims as detailed in Table 1.

A total of 10 tablets or capsules from each supplement were weighed and then crushed using a mortar and pestle to a fine powder. Each powdered supplement was prepared in DMSO (dimethyl sulphoxide) at a concentration of 10 mg/mL in a 1.5 mL capped plastic tube. Tubes were sonicated for 30 min in an ultrasonic bath (Elma Transsonic T460/H, GmbH & Co. KG, Singen (Hohentwiel), Germany) and left overnight in a carousel mixer. Then the tubes were centrifuged, and an aliquot of the supernatant was diluted in methanol from 20× to 500× depending on the claimed citrus bioflavonoid content of the supplement by the manufacturer. These solutions were then analysed by Ultra-Performance Liquid Chromatography–Mass Spectrometry (UPLC–MS/MS), (Waters Corporation, Milford, MA, USA).

### 2.2. Chemicals and Reagents

All the standards and reagents were analytical grade. Eriocitrin (98%), eriodictyol (98%), hesperidin (98%), hesperetin (98%), naringin (98%) and naringenin (98%) were purchased from Aktin Chemicals Inc. (Chengdu, China). Rutin (94%) was from Sigma-Aldrich (Castle Hill, NSW, Australia).

### 2.3. UPLC–MS/MS Analysis

Chromatography was performed using a Waters Acquity^®^ H-class UPLC on a BEH C18 column (2.1 × 100 mm × 1.7 μm) (Waters Corporation, Milford, MA, USA). The mobile phase consisted of 1.0% (*v*/*v*) acetic acid (Acetic acid OPTIMAL LC/MS grade 50 mL) (solvent A) and acetonitrile (Acetonitrile OPTIMAL LC/MS grade 4 L EcoSafPak) (solvent B). Elution consisted of an initial value of 100% of solvent A before a gradient to 40% of solvent B over 7 min. This was held for 1 min before returning to the initial conditions and re-equilibration for 3 min. The flow rate was 0.35 mL per min and the column was held at 45 °C. Injection volume was 2 μL. The UPLC was coupled to a Waters Xevo^®^ tandem mass spectrometer (Waters Corporation, Milford, MA, USA). Analyses were undertaken using multiple reaction monitoring (MRM) in negative electrospray ionisation mode, performed with a capillary voltage of 2.7 kV, and individual cone voltages and collision energies for each MRM transition, as described (Table 2). The desolvation temperature was 450 °C, nebulising gas was nitrogen at 950 L/h and cone gas was nitrogen at 50 L/h. MRM transition dwell times were 0.018 s. This is a quantitation method. It was validated using the signal-to-noise (S/N) ratio. We used a blank signal, background noise, and baseline signal through the UPLC–MS/MS.

### 2.4. Rutin Equivalence

The rutin equivalence (R_e_) for each bioflavonoid (fl) in each supplement was determined, using IC_50_ values determined previously [3], using the following formula:

R_e_(fl) = IC_50_ (rutin)/IC_50_ (fl) × mass (fl)

where

IC50 ub>50 (rutin) = Concentration of rutin to inhibit DPP-4 by 50%.
IC_50_ (fl) = Concentration of bioflavonoid to inhibit DPP-4 by 50%.
Mass (fl) = Mass of flavonoid in each tablet × daily dosing (number of tablets per day).

The total rutin equivalence for each supplement was calculated by adding the Re(fl) values for each individual bioflavonoid in that supplement.

## 3. Results

**Figure 1:**
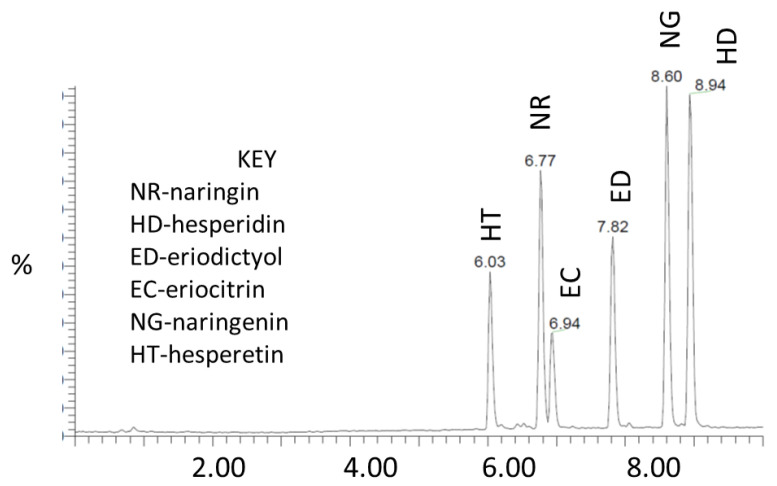
Chromatograms of the citrus bioflavonoids’ standard peaks. ER, eriocitrin; ED, eriodictyol; NR, naringin; NG, naringenin; HD, hesperidin; HT, hesperetin.

Figure 1 below shows the chromatogram for the six citrus bioflavonoids standard peaks.

**Figure 2:**
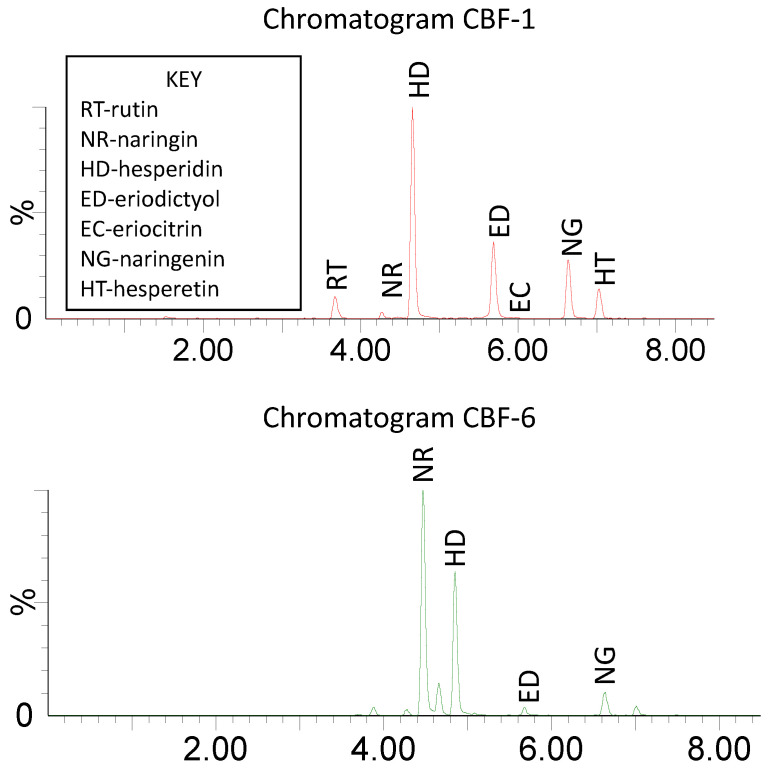
Example chromatograms of supplements CBF-1 and CBF-6, showing bioflavonoid peaks.

Examples of UPLC–MS/MS MRM chromatograms are presented in Figure 2. Supplement CBF-1 contained 15.7% bioflavonoids, dominated by hesperidin and rutin. By contrast, CBF-6 contained only 0.8% bioflavonoids, principally naringin and hesperidin. Both chromatograms demonstrated satisfactory resolution and peak shape with high sensitivity.

Table 3 details the flavonoid profile of each supplement, determined by UPLC–MS/MS analysis, expressed as % *w*/*w* of each flavonoid analysed in the supplement extract, with respect to the total flavonoid content. The final column of Table 3 describes the total flavonoid content of each supplement expressed as the % *w*/*w* of flavonoids with respect to the supplement mass.

Each manufacturer recommended a daily intake of between one and three tablets or capsules per day, as detailed in Table 4. This table also shows the daily bioflavonoid dose and the rutin equivalence values, with respect to DPP-4 inhibition, calculated for each supplement.

## 4. Discussion

The citrus bioflavonoid profile of each supplement is dependent on the citrus source of the supplement and any additional flavonoids that have been added. Of the 10 supplements, six had a hesperidin content of greater than 93%. These supplements all had undetectable levels of naringin. This profile suggests a citrus source of sweet orange (*Citrus sinensis*) [4], most likely as a by-product of orange juice production. Of the other four supplements, three had a high level of rutin (between 25% and 80%). Rutin is classified as a citrus flavonoid but it is not found in high concentration in the main commercially grown citrus fruits. The highest rutin content in citrus juice was found in grapefruit (*C. paradisi*) juice with rutin constituting 7% of the flavonoids in the juice [5]. As indicated on the product labels, rutin was added to supplements CBF-4, CBF-7 and CBF-8. The flavonoid profiles of CBF-4 and CBF-8 suggest that the citrus source of the flavonoids, besides rutin, was sweet orange, similar to the majority of the other supplements investigated. Supplement CBF-7 contained hesperidin as well as naringin which suggests a mixed citrus source that may include mostly sweet orange and some grapefruit (*C. paradisi*) or bitter orange (*C. aurantium*) [4]. The exceptional supplement of those tested was CBF-6 that contained mostly naringin (84.08%) and hesperidin (14.25%). This flavonoid profile suggested grapefruit as the main citrus source since naringin is the predominant flavonoid in grapefruit [6]. There are known interactions between flavonoids and furanocoumarins from grapefruit with various drugs, primarily due to inhibition of CYP3A4. [7]. The most affected drugs are some calcium channel antagonists, benzodiazepines, statins and cyclosporine [8]. The known adverse effects of grapefruit extract make it a poor choice for a citrus bioflavonoid supplement, particularly as the label on the product had no warning disclaimer of potential adverse drug interactions associated with grapefruit. The label also did not indicate the specific citrus source of the flavonoids, stating only that the supplement contained bioflavonoid complex from the *Citrus* species.

The supplement with the lowest content of flavonoids on a % *w*/*w* basis was CBF-6 with a value of 0.8%, which may be related to the source of the flavonoids. This contrasted with a flavonoid content of 33.3% in CBF-3. The mean (±SD) flavonoid content of all supplements was 17.0 ± 11.7. These data highlight the high degree of variability in the flavonoid content of the supplements. The label claims for the products included the content of citrus bioflavonoid extract or complex and any additional flavonoids incorporated into the supplements. From our data, it is possible to calculate the flavonoid content of citrus extract or complex for some of the supplements. For example, CBF-6, with a claimed 750 mg of citrus bioflavonoid complex, contained only 9.9 mg of flavonoids, equivalent to 1.3% of the bioflavonoid complex. By comparison, CBF-2 had a claim of 100 mg of citrus bioflavonoid extract and contained 29.1 mg of flavonoids (29.1%). The highest percentage of flavonoids in the extract or complex was in CBF-4 which, after allowing for added rutin, contained 38.6% flavonoids in its 500 mg of extract. Citrus bioflavonoid extract/complex that is used in the formulation of these citrus bioflavonoid supplements obviously varied in the degree of concentration of flavonoids. The flavonoid content of a citrus extract/complex is dependent on the citrus source used and the processing methodology which, in turn, depends on the expertise and technology available in the processing facility. We can only speculate whether the supplement manufacturers themselves know the flavonoid content of the supplements that they produce, especially since the extract/complex may be purchased from a third party. The terms flavonoid extract and flavonoid complex have little meaning without some verification of their flavonoid content. Our analysis highlight the inadequacy of supplement labelling that fails to adequately describe the actual content of flavonoids in the supplements.

Some of the supplements had their flavonoid content boosted with additional rutin (CBF-4 and CBF-7), hesperidin (CBF-9 and CBF-10) or both rutin and hesperidin (CBF-3 and CBF-8), (Table 1). From our results, it is possible to calculate the rutin content of the supplements that have had rutin added to check the label claims made for those supplements. For CBF-4, we determined 87 mg of rutin per tablet, versus the claim of 100 mg of rutin added. The rutin contributed by the citrus bioflavonoid extract is relatively small and was not considered. For CBF-3, the supplement contained only 9.8% of the expected rutin content. For CBF-7 and CBF-8 the respective values were 77% and 71% of the expected rutin content. It is evident that CBF-3, in particular, contained significantly less rutin than would be expected based on the claim indicated on the label. The discrepancy was greater than 90%, which reflected a major deficiency in product formulation quality control.

The majority of rutin that is commercially available is sourced from *Sophora japonica* or from *Fagopyrum esculentum* (buckwheat) rather than from citrus sources [9,10,11]. It is not evident from the labels of the supplements from which source the rutin was obtained. China is currently the largest supplier of rutin globally and most of the rutin appears to be produced from *S. japonica*.

It is more difficult to assess the quantity of hesperidin added to supplements because hesperidin is a major constituent of sweet orange flavonoids and therefore there are two possible sources for hesperidin in the supplements. However, in the case of CBF-10, the total content of hesperidin was determined to be 107 mg per tablet and the label claim was that each tablet contained 245 mg of hesperidin in addition to the hesperidin content from 700 mg of citrus bioflavonoid complex. In this case, it is clearly evident that the added hesperidin was less than 46% of the quantity claimed by the manufacturer.

It is not possible from this study to determine the reasons for the discrepancies between label claims of added flavonoids and the actual content of flavonoids. Presumably it may be due to the quality of the flavonoids supplied to the supplement manufacturer and/or due to inadequate quality control on the part of the manufacturer. The major discrepancies identified in this study were in products manufactured in the USA. The poor quality of some of the USA products may reflect the level of regulatory oversight of complementary healthcare products in this jurisdiction. Additionally, the citrus source of bioflavonoids should be disclosed on the product label for better health benefits and to reduce risk of adverse side effects.

The potential of the citrus bioflavonoid supplements to inhibit DPP-4 and thereby to improve glycaemic regulation in diabetic and prediabetic individuals depends on the daily dose of flavonoids and the flavonoid profile in the supplement. Each flavonoid varies in its inhibitory potency towards DPP-4, as measured by its IC_50_ through in vitro assay. We have previously determined the IC_50_ values of the flavonoids [3], which made it possible to express each flavonoid as an equivalent amount of rutin, and then to determine the daily dose of each supplement as a rutin equivalent, as detailed in Table 4. From the data in Table 4 it is evident that there was a wide range in the calculated daily dose rutin equivalence for the supplements. CBF-6 had the lowest value of 1.9 mg of rutin equivalent and CBF-7 had the highest value of 400 mg of rutin equivalent. CBF-7 contained the highest concentration of added rutin (claimed as 500 mg, determined as 387 mg) which is why it had the highest rutin equivalence of all the supplements. This gives supplement CBF-7 the greatest potential for beneficial effects in glycaemic regulation, based on data from in vitro analyses of DPP-4 inhibition. The mean (±SD) value of rutin equivalence for the 10 supplements was 95 ± 124 mg, which demonstrates a high degree of variability in rutin equivalence between the supplements. The median value of rutin equivalence was 49.7 mg, so half of the supplements had a rutin equivalence of less than 50 mg. By comparison a glass of freshly squeezed orange juice (250 mL) has a rutin equivalence of less than 10 mg [5].

Although we determined the rutin equivalence of each flavonoid supplement, based on in vitro IC_50_ data, to enable a comparison of supplements, the actual in vivo effects of the supplements may not be simply represented by the calculated rutin equivalence. Flavonoids have generally low and variable bioavailability in humans, with some glycosides requiring hydrolysis by intestinal bacteria to enable absorption [12,13]. Catabolism of flavonoids to simpler phenolic acids, that are then absorbed from the intestine, is also an important pathway of absorption [13]. Flavonoids may also be extensively modified post absorption to various metabolites [14]. While the relatively high (compared with gliptins) IC_50_ determined for rutin with respect to DPP-4 inhibition was 296 mg/L [3], the actual effect of ingestion of citrus bioflavonoids may be exerted through the action of metabolites or catabolites that may have more potent inhibitory activities. The effects of citrus bioflavonoids on DPP-4 activity in vivo, and therefore the actual benefits of the supplements for patients with compromised glycaemic control, cannot be determined other than through a clinical trial.

In summary, supplement flavonoid content varied from 0.8 to 33.3% *w*/*w*, a variation of more than 40 times. The recommended daily dose varied from 19 mg to 560 mg of flavonoids. DPP-4 inhibition potential of the supplements varied from 1.9 mg to 400 mg (rutin equivalence).

## 5. Conclusions

The main citrus bioflavonoid in 9 of the 10 supplements was hesperidin, its probable source being oranges. One supplement had naringin as the main citrus bioflavonoid, suggesting a grapefruit source with the potential for serious drug interactions. Analysis of the supplements with added rutin suggested levels of rutin lower than the label claims for the products. Some supplements possessed much lower DPP-4 inhibitory potential than others. Actual DPP-4 inhibition needs to be assessed by in vivo studies.

## Figures and Tables

**Table 1:** Commercial supplements investigated in this study and their claimed citrus bioflavonoid content from crude citrus (extract or complex) and additional constituents.

Supplements	Origin	Type	Sample Access Date	Citrus Bioflavonoids	+Rutin (mg)	+Hesperidin (mg)	+Vit C (mg)
CBF-1	USA	Tablet	10 June 2019	1000 mg complex	0	0	0
CBF-2	AUS	Tablet	10 June 2019	100 mg extract	0	0	1000
CBF-3	USA	Tablet	11 June 2019	500 mg complex	75	75	0
CBF-4	AUS	Tablet	11 June 2019	500 mg extract	100	0	500
CBF-5	AUS	Tablet	12 June 2019	500 mg extract	0	0	500
CBF-6	USA	Capsule	12 June 2019	750 mg complex	0	0	0
CBF-7	USA	Tablet	13 June 2019	550 mg complex	500	0	0
CBF-8	USA	Tablet	13 June 2019	900 mg complex	100	37.5	0
CBF-9	CAN	Capsule	14 June 2019	500 mg extract	0	0	150
CBF-10	USA	Capsule	14 June 2019	700 mg complex	245	0	500

**Table 2:** Quantification MRM (Mass Spectrometry) and electrospray ionisation parameters of each analyte and LLoQ (lower limit of quantitation) based on 10× S/N [3].

Analytes	Rt (min)	Precursor (*m*/*z*)	Products (*m*/*z*)	Cone V	Col V	LLoQ (pg/mL)
Rutin (94%)	3.65	609.4	300.2	49	36	220
Eriocitrin (98%)	3.67	595.4	287.2	49	22	130
Naringin (98%)	4.53	579.4	271.2	49	32	280
Hesperidin (98%)	4.71	609.4	301.2	49	24	30
Eriodictyol (98%)	5.87	287.2	151.1	49	15	70
Naringenin (98%)	6.91	271.2	151.1	49	18	100
Hesperitin (98%)	7.15	301.2	164.1	49	25	430

Rt, retention time; Col, column.

**Table 3:** Citrus bioflavonoids content of commercial supplements.

Supplements	RT %	EC %	ED %	NR %	NG %	HD %	HT %	Total % *w*/*w*
CBF-1	0.93	0.18	1.99	0.00	1.47	93.8	1.65	15.7
CBF-2	0.4	0.4	0.27	0.00	0.09	98.3	0.54	1.9
CBF-3	3.32	0.83	0.21	0.00	0.06	95.2	0.37	33.3
CBF-4	31.1	0.04	0.04	0.00	0.13	67.9	0.72	22.1
CBF-5	0.32	1.66	0.01	0.00	0.1	97.4	0.47	6.1
CBF-6	0.00	0.00	0.4	84.1	1.27	14.25	0.00	0.8
CBF-7	79.7	0.00	0.09	0.26	0.00	19.9	0.00	32.1
CBF-8	25.4	0.00	0.01	0.00	0.02	74.5	0.11	21.5
CBF-9	0.32	0.39	0.00	0.00	0.18	98.8	0.31	24.4
CBF-10	0.04	0.00	0.00	0.00	0.02	99.7	0.23	11.7

RT, rutin; EC, eriocitrin; ED, eriodictyol; NR, naringin; NG, naringenin; HD, hesperidin; HT, hesperetin; % *w*/*w*-total bioflavonoids with respect to supplement mass.

**Table 4:** Recommended daily dose of each supplement expressed as rutin equivalence with respect to DPP-4 inhibition.

Supplements	Daily Dose (mg)	Daily Flavonoid Dose (mg)	Rutin Equivalence (mg)
CBF-1	1442 (1 tablet)	226	30.6
CBF-2	4598 (3 tablets)	88	11.2
CBF-3	1329 (2 tablets)	443	68
CBF-4	2532 (2 tablets)	560	222
CBF-5	2864 (2 tablets)	174	22.1
CBF-6	2468 (2 tablets)	19	1.9
CBF-7	1513 (1 tablet)	486	400
CBF-8	1303 (1 tablet)	280	96.9
CBF-9	1915 (3 tablets)	467	59.4
CBF-10	2747 (3 tablets)	321	40
